# Advanced Fluorescence Protein-Based Synapse-Detectors

**DOI:** 10.3389/fnsyn.2016.00016

**Published:** 2016-06-30

**Authors:** Hojin Lee, Won Chan Oh, Jihye Seong, Jinhyun Kim

**Affiliations:** ^1^Center for Functional Connectomics, Korea Institute of Science and TechnologySeoul, South Korea; ^2^Neuroscience Program, Korea University of Science and TechnologyDaejeon, South Korea; ^3^Center for Diagnosis Treatment Care of Dementia, Korea Institute of Science and TechnologySeoul, South Korea

**Keywords:** fluorescent protein sensors, synaptic connectivity, synapses, gene delivery, mapping and localization, light microscopy

## Abstract

The complex information-processing capabilities of the central nervous system emerge from intricate patterns of synaptic input-output relationships among various neuronal circuit components. Understanding these capabilities thus requires a precise description of the individual synapses that comprise neural networks. Recent advances in fluorescent protein engineering, along with developments in light-favoring tissue clearing and optical imaging techniques, have rendered light microscopy (LM) a potent candidate for large-scale analyses of synapses, their properties, and their connectivity. Optically imaging newly engineered fluorescent proteins (FPs) tagged to synaptic proteins or microstructures enables the efficient, fine-resolution illumination of synaptic anatomy and function in large neural circuits. Here we review the latest progress in fluorescent protein-based molecular tools for imaging individual synapses and synaptic connectivity. We also identify associated technologies in gene delivery, tissue processing, and computational image analysis that will play a crucial role in bridging the gap between synapse- and system-level neuroscience.

## Introduction

The synapse is the primary site for neurons to make functional contacts for exchanging information. The term “synapse”, meaning conjunction in Greek (synapsis = together + to fasten), was coined in 1897 by the eminent physiologist Charles Scott Sherrington (Nobel Laureate 1932). But the idea that synapses play critical roles as dynamically polarized, communication contacts was proposed by Santiago Ramon y Cajal (Nobel Laureate 1906). Since then, the synapse as a structural and functional communication unit has been in the spotlight of neuroscientific inquiry (Cowan et al., 2001). In fact, many studies have demonstrated that synaptic events, such as changes in molecular composition, structure, efficacy, and potentiation, play important roles in brain functions including memory formation, perception, and other complex behaviors (Tsien et al., 1996; Markram et al., 1997; Malinow and Malenka, 2002; Russo et al., 2010; Caroni et al., 2014). An important focus has been to visualize the synapse and to measure its activity. In 1954, DeRobertis and Palay first observed synapses independently by electron microscopy (EM) and George Gray suggested there may be different types of synapses, i.e., excitatory and inhibitory (De Robertis and Bennett, 1955; Palay and Palade, 1955; Palay, 1956; Gray, 1959). EM provides enough resolution for nanometer-scale imaging of the synaptic ultrastructure, something that cannot be achieved by light microscopy (LM) because of its diffraction limit. However, despite recent advances that have reduced the time needed for image acquisition and reconstruction, EM remains inherently time-consuming, labor-intensive, and volume-limited for large neural circuits.

With the recent engineering of fluorescent proteins (FPs) and new developments in light-favoring tissue clearing and advanced optical methods, LM is rising as a potent alternative tool for investigating individual synapses in the context of neural networks (Gray, 1959; Keller et al., 2008; Kim et al., 2012; Tomer et al., 2012; Chung et al., 2013; Richardson and Lichtman, 2015). Imaging the synapse with LM by using newly engineered FPs tagged to synaptic proteins or targeted to synaptic structures enables fine-resolution illumination of synaptic anatomy and function in large neural circuits, possibly in real-time. In this review, we describe recent FP-based molecular tools for imaging individual synapses and synaptic connectivity in the contexts of single- and dual component synaptic detection. We also identify crucial technologies: gene delivery of molecular synapse detector; tissue clearing for whole-brain imaging; and computational analysis, whose parallel development has potential to bridge synaptic sensor engineering and systems neuroscience. We end by proposing a scheme of technological integration for synaptic neuroscience at the systems level.

## Single Component Synaptic Detection

**Figure 1:**
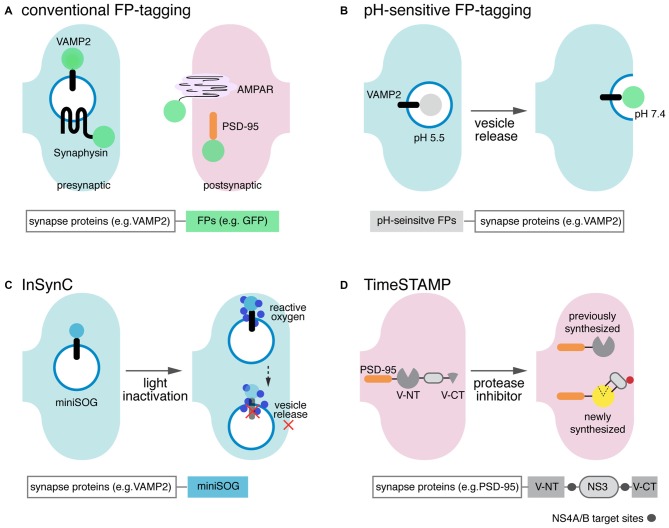
**Scheme of single component synapse detectors. (A)** Graphical depiction of the conventional green fluorescent protein (GFP) tagging scheme and summary of the expressed carrier-sensor construct. GFP (green circle) and other fluorescent proteins (FPs) are directly tagged to synaptic proteins in the presynaptic terminal or postsynaptic spines. Major targets for tagging include presynaptic vesicle proteins (e.g., vesicle-associated membrane protein 2 (VAMP2) and synaptophysin), postsynaptic receptors (e.g., α-amino-hydroxy-5-methyl-4-isoxazolepropionic acid receptor, AMPAR) and postsynaptic density protein-95 (PSD-95). **(B)** pH-sensitive FP mutants are fused to synaptic vesicle membrane proteins, such as VAMP2, to visualize vesicle secretion/recycling and neurotransmission. A pH-sensitive GFP variant, pHluorin does not fluoresce (gray circle) when inside the acidic chemical environment of the synaptic vesicle, but becomes highly fluorescent (green circle) when the vesicle is released and exposed to the neutral extracellular environment. **(C)** Inhibition of Synaptic Release with CALI (InSynC): attached to target SNARE proteins, molecular actuators such as mini small singlet oxygen generator (miniSOG; light blue filled-in circle) selectively inactivate specific synaptic proteins that regulate vesicle release and other synaptic events. When illuminated with blue light, miniSOG stimulates generation of reactive oxygen species (small, dark blue filled-in circles), which then oxidizes susceptible amino acid residues in target vesicle proteins and deactivates protein functions.** (D)** TimeSTAMP effectively tracks spatiotemporally controlled protein synthesis and trafficking in living neurons. In the presence of a membrane-permeable protease inhibitor, NS3 protease (gray oval) activity is inhibited, and Venus C-terminus (Venus CT) and Venus N-terminus (Venus NT) reconstitute as fluorescent Venus (yellow circle). Reconstituted Venus accumulates in the postsynaptic spine, the trafficking destination of the fused PSD-95. When the protease inhibitor is present, however, NS3 protease cleaves the protease target sites (gray circles flanking NS3), preventing Venus reconstitution.

The discovery of green fluorescent protein (GFP) and its derivatives revolutionized the visualization of biological phenomena, including the individual synapse and its functions. GFP and other FPs are relatively inert and small (27 kDa) and can be used to tag synaptic proteins while minimally interfering with their normal functions. In fact, the distribution, trafficking, and physiological changes of synaptic proteins caused by neural activity became evident in the last two decades, largely through observations of synaptic proteins, such as synaptophysin (Li and Murthy, 2001) vesicle-associated membrane protein 2 (VAMP2; Ahmari et al., 2000), postsynaptic density protein-95 (PSD-95; Nelson et al., 2013) calcium/calmodulin-dependent kinase II (CaMKII; Shen et al., 2000), α-amino-hydroxy-5-methyl-4-isoxazolepropionic acid receptor (AMPAR; Zamanillo et al., 1999) and so forth, that had been tagged with FPs. Recently, sophisticated molecular engineering has allowed even more precise and detailed visualization of synaptic structure, composition, and physiology (Chen et al., 2014; Fortin et al., 2014; Figure 1, Table 1).

**Table 1:** **Summarized methods of single and dual component synapse detection**.

	Single component			Dual component				
	Synaptic release-detecting FPs	InSynC	TimeSTAMP	GRASP	mGRASP	Syb:GRASP XRASP	SynView	ID-PRIME
**Detection/working module**	pH-sensitive FPs: pHluorin, pHTomato	miniSOG-generated reactive oxygen	Reconstitution of VenusNT and VenusCT	Reconstitution of spGFP1-10 and spGFP11	Reconstitution of spGFP1-10 and spGFP11	Reconstitution of spXFP1-10 and spXFP11	Reconstitution of spGFP1-10 and spGFP11	Enzymatic interaction of LpIA and LAP
**Synaptic targeting module**	Synaptophysin, VAMP2, PSD-95, CaMKII, vGLUT1, mGluRs	Synaptophysin, VAMP2	PSD-95, CaMKII, Neuroligin	Human CD4, PTP-3A, nlg-1	Rat neurexin1-β, mouse neuroligin-1	Synaptobrevin, human CD4	Rat neurexin-1β, rat neuroligin-1, 2	Human neurexin-3β, mouse neurexin-1β, rat neuroligin-1
**Model Systems**
*In vitro*	HEK, primary cultured neurons, organotypic slices	Primary cultured neurons, organotypic slices	HEK, primary cultured neurons	N/A	N/A	HEK	HEK, COS-7, primary cultured neurons	HEK, primary cultured neurons
*In vivo*	N/A	N/A	N/A	*C.elegans, Drosophila*	Mouse	*Drosophila*	N/A	N/A
**Remarks**	Tracking of presynaptic vesicle release, postsynaptic receptor trafficking	Optically inhibiting synaptic release with good spatial resolution	Optical pulse-chase labeling of synaptic proteins with high spatiotemporal resolution	*in vivo* synaptic detection in invertebrate circuits	*in vivo* synaptic detection in vertebrate circuits	Activity-dependent labeling of multiple synaptic inputs	Synapse labeling by direct interaction of neurexin-neuroligin	Enzyme-based visualization of direct interaction of neurexin-neuroligin
	Lacks *in vivo* application with quantitative analysis	Lacks physiologically relevant temporal resolutions due to slow recovery	Awaits mammalian *in vivo* application	Limited to apply mammalian system	Limited to detect functional synaptic connections	Limited to apply mammalian system, limited temporal resolutions	Lacks *in vivo* verification and application	Requires separate introduction of exogenous ligase and antibody-fluorophore conjugates
**References**	Miesenböck et al. (1998), Sankaranarayanan and Ryan (2000), Kopec et al. (2006), Voglmaier et al. (2006) and Li and Tsien (2012)	Lin et al. (2013)	Lin et al. (2008) and Butko et al. (2012)	Feinberg et al. (2008), Gordon and Scott (2009), Gong et al. (2010), Park et al. (2011), Yuan et al. (2011), Fan et al. (2013), Pech et al. (2013) and Gorostiza et al. (2014)	Feng et al. (2012), Kim et al. (2012) and Druckmann et al. (2014)	Karuppudurai et al. (2014), Frank et al. (2015), Macpherson et al. (2015) and Li et al. (2016)	Tsetsenis et al. (2014)	Liu et al. (2013)

### pH-Sensitive FPs for Visualizing Vesicle Release: pHluorin, pHTomato, and pHuji

Although previous, straightforward, FP-based detection of synaptic distribution revealed many important details of synaptic physiology, synaptic vesicle release/recycling and neural activity-driven changes in membrane-bound synaptic proteins are difficult to be detected by regular FP-tagging. Special sensors of vesicle secretion and neurotransmission have been developed by linking vesicle membrane proteins with pH-sensitive mutants of GFP called pHlourin (Miesenböck et al., 1998). The fluorescence intensity of pHluorin largely depends on the pH of its biochemical environment: in acidic environments with pH below 6.5, pHluorin is mostly nonfluorescent in 480 nm of light illumination, but becomes highly fluorescent in neutral environments with pH around 7.4. This special feature of pHluorin was achieved by several mutations on residues important for the proton-relay of tyrosine 66 in the chromophore (S147D, N149Q, T161I, S202F, and Q204T). These amino acid substitutions, which set the pKa of pHluorin to around 7.0, can facilitate the pH-dependent switching of the electrostatic environment of the chromophore, allowing the pH-dependent changes in fluorescent intensity (Sankaranarayanan and Ryan, 2000). When pHluorin is fused to presynaptic vesicle proteins such as VAMP2 (Miesenböck et al., 1998), synaptophysin (Zhu et al., 2009), and vesicular glutamate transporter (vGluT; Voglmaier et al., 2006), the release and recycle of synaptic vesicles can be monitored as the pH inside the synaptic vesicles (~5.5) transitions to the pH of the extracellular environment (~7.4). Thus, by tracking changes in pHluorin fluorescence intensity, one can detect real-time presynaptic exocytosis in living, active neurons. Similarly, postsynaptic endo-/exo-cytosis and related receptor dynamics can be visualized by pHluorin-fused mGluRs, for instance (Pelkey et al., 2007).

More recently, red pH-sensitive FPs such as pHTomato (Li and Tsien, 2012) and pHuji (Shen et al., 2014) have been developed, allowing for the simultaneous monitoring of multiple synaptic activities when combined with the green GFP-based sensors (e.g., GCaMP). pHTomato (pKa ~ 7.8) was derived from mStrawberry by introducing six mutations (F41T, F83L, S182K, I194K, V195T, and G196D). Synaptophysin-fused pHTomato (sypHTomato) has been shown to be suitable for simultaneously monitoring the fusion of synaptic vesicles and, when paired with GCaMP3, postsynaptic Ca^2+^ changes in living neurons. In addition, multiple synaptic events have been successfully measured by using sophisticated combinations of these pH-sensitive FP-fused synaptic proteins and spectrally distinct variants of optogentic stimulators (ChR2-T2A-vGluT-pHlourin and VChR1-T2A-synpHTomato). However, the pH-sensitivity of pHTomato is relatively low (3-fold change in pH 5.5–7.5). Thus, a new red pH-sensitive FP, named pHuji, has been more recently derived from mApple (Shaner et al., 2008) by including a K163Y mutation, resulting in high pH sensitivity (20-fold change in pH 5.5–7.5). The use of different colored pH-sensitive FPs together with a Ca^2+^ indicator and/or a spectrally distinct optogenetic modulator offers a new promising readout system for complex, coordinated synaptic events.

### Inhibition of Synaptic Release with CALI (InSynC)

Beyond merely visualizing the distributions and endo-/exocytosis of synaptic proteins by tagging them with FPs and their pH-sensitive variants, genetically encoded chromophore-assisted light inactivation (CALI) has been developed to selectively inactivate specific synaptic protein functions that regulate synaptic events such as synaptic release (Lin et al., 2013). CALI is based on light-induced generation of reactive oxygen and the consequent inactivation of nearby attached synaptic proteins. Its original agents were synthetic chromophores for example malachite green (Jay, 1988), fluorescein (Beck et al., 2002), FlAsH (Marek and Davis, 2002), ReAsH (Tour et al., 2003), and eosin (Takemoto et al., 2011) and FPs such as KillerRed (Bulina et al., 2006). To precisely inhibit synaptic release with improved target specificity and inactivation efficiency compared to these CALI agents, inhibition of synaptic release with CALI (InSynC) has been recently developed using a newly engineered flavoprotein called mini small singlet oxygen generator (miniSOG), fused with the SNARE proteins VAMP2 and synaptophysin (Lin et al., 2013). miniSOG was originally derived from the light, oxygen, and voltage 2 (LOV2) domain of phototropin, a blue light photoreceptor (Shu et al., 2011). Under blue light illumination, this photoreceptor binds to and excites flavin mononucleotide (FMN), which then functions as an oxygen generator in cells. miniSOG contains the single amino acid substitution of FMN-binding residue Cys426 to Gly on the LOV2 domain, allowing for more efficient energy transfer to FMN, and contains further mutations for enhanced brightness. When illuminated by blue light, synaptic proteins can be selectively inactivated by miniSOG-mediated oxidization of susceptible residues such as tryptophan, tyrosine, histidine, cysteine, and methionine. InSynC by miniSOG can selectively inhibit vesicle release at individual synapses *in vitro* and *in vivo* thanks to the high efficiency of its light-induced oxygen generation, independence of exogenous cofactors, and small size (106 residues, 14 kDa; Lin et al., 2013). However, further engineering is required for investigating the functional dynamics of synaptic circuits with physiologically relevant temporal resolutions, as inactivation by InSynC persists relatively long (~1 h) after light stimulation.

### Time-Specific Tagging for the Age Measurement of Proteins (TimeSTAMP)

Another new strategy beyond merely visualizing synaptic proteins by tagging with FPs is time-specific tagging for the age measurement of proteins (TimeSTAMP; Lin et al., 2008). As spatiotemporally controlled protein synthesis and trafficking are critical for synaptogenesis, synaptic connectivity, and long-lasting changes in synapses, TimeSTAMP is beneficial for tracking important, newly synthesized synaptic proteins such as PSD-95 and CaMKII in living neurons. TimeSTAMP is a drug-controllable, time-specific tagging strategy based on the hepatitis C virus NS3 protease and its cell-permeable inhibitor BILN-2061. PSD-95-GFP, for example, was fused to NS3 protease flanked by NS4A/B target sites and the C-terminal HA tag (PSD-95-GFP-TS-HA). In the absence of the specific inhibitor BILN-2061, NS3 protease cleaves the NS4A/B target sites allowing the C-terminal HA-tag to be cleaved from PSD-95-GFP and degraded; but drug application will allow the HA-tag to be accumulated. This allows distinguishing between newly and previously synthesized PSD-95 by measuring HA/GFP signals at a time defined by the drug application. For the TimeSTAMP strategy, the NS3 protease domain was chosen because it is small (19 kDa) and monomeric, specific to its substrate, and not cytotoxic, and most of all, NS3 shows high selectivity and efficiency of its cell-permeable inhibitor.

Although the first version of TimeSTAMP has worked successfully in primary hippocampal neurons to track PSD-95 accumulation during synaptic growth, and in *Drosophila* for whole-brain mapping of newly synthesized CaMKII, it required *post hoc* immunostaining against epitope tags, limiting the benefits of pulse-chase labeling through time. Thus, TimeSTAMP2 has been introduced, replacing HA-tag with split-fluorescence proteins (e.g., Yellow fluorescent protein, YFP; Orange fluorescent protein, OFP). In fluorescent TimeSTAMP2, for instance, Venus yellow fluorescent protein is separated by an NS3 domain flanked by NS4A/B target sites into VenusNT (1-158 aa) and VenusCT (159-238 aa) for drug-dependent fluorescence. In the absence of BILN-2061, VenusCT is cleaved and degraded by the active NS domain resulting in no yellow fluorescence, while VenusNT and CT are reconstituted as fluorescent forms after drug application. This allows optical pulse labeling of synaptic proteins such as PSD-95 and Neuroligin with a drug-defined temporal resolution. Additionally, photo-oxidizing TimeSTAMP using miniSOG inserted into TS:YFP can be used to visualize new proteins at an EM-based ultrastructural level (Butko et al., 2012).

## Dual Component Synaptic Detection

**Figure 2:**
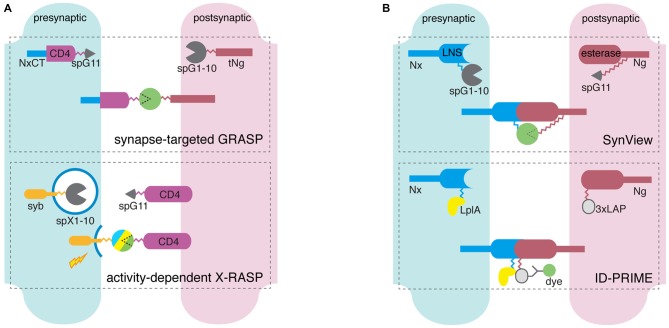
**Scheme of dual component synapse detectors. (A)** GFP reconstitution-dependent molecular tools detect pre- and postsynaptic membrane apposition through the reconstitution of spGFP fragments each attached to the extracellular ends of membrane protein carriers. Synapse-targeted GFP Reconstitution Across Synaptic Partners (GRASP) utilizes a presynaptic component of spGFP11 (spG11, circular wedge) linked to a CD4-neurexin-1β C-terminus (NxCT) fusion through flexible peptide linkers (gray zigzag), and a postsynaptic component of spGFP1-10 (spG1-10) and truncated neuroligin-1 (tNg) connected with a linker. SpG11 and spG1-10 unite at synapses, not random membrane contacts. In activity-dependent X-RASP the presynaptic split-XFP1-10 (spX1-10) is fused to SNARE proteins such as synaptobrevin (syb), and unites with CD4-bound spG11 when presynaptic activity triggers vesicle release. **(B)** Neurexin (Nx)-neuroligin (Ng) interaction-dependent molecular tools detect individual synapses through fluorescent protein (FP)-based (SynView) and enzymatic reaction-based (Interaction-Dependent Probe Incorporation Mediated by Enzymes, ID-PRIME) direct visualization of Nx-Ng interaction. In SynView, spG11 is inserted between the esterase and proximal extracellular domain of Ng, and spG1-10 in the middle of the LNS domain of Nx. GFP reconstitutes when Nx and Ng unites. ID-PRIME uses the identical set of synaptic protein carriers, but tandem LAP tags (3xLAP) and lipoic acid ligase A (LplA) are attached to C-terminus of Ng esterase domain and Nx LNS domain, respectively. Nx-Ng interaction initiates LplA-mediated lipoic acid tagging to 3xLAP, and signals useful for imaging originate from antibody-fluorophore conjugates.

Thus far, we have described methods for detecting synapses by labeling single components, either pre- or post-synaptic. Although these single-component tools allow for detecting, measuring, and manipulating synaptic structures and activities, they do not address the critical fact that synapses are bilateral microstructures involving *both* presynaptic terminal and postsynaptic density. For reliable synapse detection, therefore, several recent studies introduced new methods for labeling synaptic interactions between pre- and post-synaptic components, such as using split-FPs or enzymes to label neurexin-neuroligin interactions. Here we review dual-component methods using handshaking-like transmembrane molecular interaction across the synaptic cleft, with particular attention to GFP Reconstitution Across Synaptic Partners (GRASP; Figure 2, Table 1).

### Mammalian GFP Reconstitution Across Synaptic Partners (mGRASP)

To detect particular synaptic connections with LM, the Mammalian GFP Reconstitution Across Synaptic Partners (mGRASP) technique takes advantage of the complementarity of two non-fluorescent split-GFP fragments, each of which is tethered specifically to the pre- and postsynaptic membrane, respectively (Kim et al., 2012). When two neurons, each expressing one of the fragments, are closely opposed across a synaptic cleft, the split fragments are reconstituted as fluorescent GFP in that location. This dual component synapse detection bypasses the Abbe’s diffraction limit and allows relatively rapid and accurate synapse mapping with LM. This method takes advantage of the fast folding kinetics and stability after maturation of superfolder GFP (Pédelacq et al., 2006), split into two fragments, namely spGFP1-10 (first 214 residues comprising ten β barrels) and spGFP11 (16 residues, 11th β-barrel strand). The GRASP technique was initially implemented in *C. elegans* (Feinberg et al., 2008; Park et al., 2011) and later in *Drosophila* (Gordon and Scott, 2009; Fan et al., 2013; Gorostiza et al., 2014). In the original GRASP constructs, membrane carriers (human CD4) for split-GFP fragments were not synapse-specific—fluorescence could arise wherever any membranes expressing fragment pairs were closely apposed. This became a critical issue for mammalian synapse detection because the mammalian nervous system contains much more compactly intermingled neurites than the invertebrate nervous system.

In a previous study, we engineered and successfully applied mGRASP for mapping fine-scale synaptic connectivity in the mouse brain (Kim et al., 2012). The primary features of mGRASP include targeting specific synapses, and matching the approximately 20 nm-wide synaptic cleft without gross changes in endogenous synaptic organization and physiology. Based on published sequences from NCBI, we generated synapse-specific chimeric carriers. Both the pre- and postsynaptic mGRASP components share a common six-subcomponent framework: (1) a signal peptide; (2) a split-GFP fragment; (3) an extracellular domain; (4) a transmembrane domain; (5) an intracellular domain; and (6) a fluorescent protein for neurite and soma visualization. The presynaptic mGRASP component consists of the signal peptide of nematode β-integrin (PAT-3, residues 1–29), GFP β-strand 11 (spGFP11, 16 residues), two flexible GGGGS linkers, the extracellular and transmembrane domains of human CD4-2 (residues 25–242). Rat neurexin-1β (residues 414–468) containing the PDZ-binding motif constitutes the intracellular domain for maintenance of correct localization at the presynaptic site. mCerulean is fused to the intracellular end of the presynaptic mGRASP to visualize axonal expression. The postsynaptic mGRASP component is based primarily on mouse neuroligin-1, which interacts with presynaptic adhesion proteins including β-neurexins, and mediates the formation and maintenance of synapses between neurons. To prevent nonspecific synaptogenesis through interactions with its endogenous partner, neurexin, the extracellular esterase domain of neuroligin is deleted in the main skeleton of the postsynaptic mGRASP. Similar to presynaptic mGRASP, post-mGRASP is composed a signal peptide from the esterase-truncated neuroligin-1 (residues 1–49), GFP β-strand 1-10 (spGFP1-10, 648 residues), the extracellular, transmembrane, and intracellular regions of neuroligin (71, 19, and 127 residues, respectively), followed by the self-cleavable 2A peptide-fused dTomato for visualizing postsynaptic neuronal morphology. This optimized mGRASP enabled the comprehensive synaptic connectivity mapping of hippocampal CA3-CA1 and identified spatially structured patterns of synaptic connectivity (Druckmann et al., 2014). It is important to note that brain-wide synapse detection for comprehensive fine-scale mapping becomes achievable not only by advanced molecular engineering to label the synapse such as mGRASP, but also by appropriately engineered computational analysis (Feng et al., 2012, 2015).

Current GRASP technology has proved to be a tool suitable for rapid and accurate mapping of synaptic connectivity in nematode (Feinberg et al., 2008), fruit fly (Gordon and Scott, 2009), and mouse (Kim et al., 2012; Druckmann et al., 2014). Yet, further improvements to GRASP such as multi-colored FPs for analyzing convergent synaptic inputs, various carriers for neural activities, and tailored computational analyses will provide a clear overview of complex synaptic connectivity and its operation. We next discuss recent efforts in these directions.

### Engineering FPs for Multi-Color GRASP

A neuron oftentimes receives multiple inputs from different presynaptic neurons of distinct cell types and/or various brain areas. For comprehensive mapping of complex synaptic circuits, multiple synaptic detection is beneficial, as described earlier in the section describing pH-sensitive FPs. The current version of GRASP restricts convergent synaptic mapping because it relies on only a single pair of spGFP fragments such that spectral overlap of its signals hinders simultaneous imaging with previously well-established GFP-based tools such as GCaMP. Therefore, the multi-color GRASP (XRASP) components have been developed recently (Macpherson et al., 2015; Li et al., 2016). Given that Cyan fluorescent protein (CFP), GFP, and YFP have identical structures except for several residues in the chromophore located in beta barrels of 1-10, C-RASP and Y-RASP were generated by color-shifting mutations in spGFP1-10 (Y66W, S72A, F145A*, N146I, and H148D for C-RASP, *additional mutation in Li et al., 2016, T65G, V68L, S72A and T203Y for Y-RASP) paired with the unaltered spGFP11 (Li et al., 2016). Multi-color GRASP (XRASP) was tested *in vivo* in several circuits, including the Kenyon cells of the mushroom body, and projection neurons of the thermosensory and olfactory systems in the fruit fly. Reconstructed CFP and YFP signals showed minimal spectral overlap with the GRASP emission and excitation spectra. Extended choices of multi-color GRASP (XRASP) will allow simultaneous imaging of multiple, convergent connectivity and functional activity. However, more red-shifted XRASP is required for *in vivo* 2-photon imaging together with Ca^2+^ indicators and for fine-scale synapse labeling from multiple inputs within the single neuron, because it has proved difficult to separate CFP/GFP/YFP signals in the complex mammalian nerve system.

### Engineering Carriers for Activity-Dependent GRASP

To understand functional organizations underlying complex brain functions that go beyond structural connectivity patterns, it will be essential to identify active synaptic connectivity at defined times and conditions. For the activity-dependent GRASP system, synaptobrevin (syb), a key constituent of synaptic vesicle membrane, was used as a synaptic carrier instead of constantly membrane-targeted carriers in the previous GRASP systems. The straightforward fusion of syb and spGFP1-10 (syb:spGFP1-10) with the original CD4:spGFP11 can together form activity-dependent GRASP, called the syb:GRASP system. Given activity-dependent interactions of syb with SNARE-SM protein complex triggering synaptic vesicle release, syb:GRASP showed preferential labeling of active synapses as a boost of GRASP fluorescence signals in well-studied thermosensory and olfactory circuits in the fruit fly (Macpherson et al., 2015). This new strategy for mapping active synaptic connectivity might expedite the mapping of functional connectivity at the synapse level in a way previously achieved only by difficult combinations of Ca^2+^ imaging and exhaustive EM reconstruction (Bock et al., 2011; Briggman et al., 2011). Yet, these new techniques raise basic concerns about possible side effects caused by the overexpression of key synaptic vesicle proteins, and need further optimization before they can be applied to mammalian networks.

### FP- and Enzyme-Based Visualization of Neuroligin-Neurexin Interaction

An alternative way to detect synapses has been suggested by imaging neurexin-neuroligin interactions. Presynaptic neurexin and postsynaptic neurolign are transmembrane adhesion proteins and their interaction at the synaptic cleft has been believed to be a key process for synaptic formation, maintenance, and connectivity (Li and Sheng, 2003; Graf et al., 2004; Chen et al., 2010; Krueger et al., 2012). Therefore, it is thought that identifying sites of neurexin-neuroligin interactions could provide selective visualization of synapses. Similar to the GRASP approaches, this method, based on neuroligin-neurexin interactions using splitGFP, is called SynView. It is composed of spGFP1-10-inserted neurexin-1β between residues N275-D276 or A200-G201 for the presynaptic component, and the split-GFP11-inserted neuroligin-1 in the C-terminal end of the extracellular esterase (between Q641-Y642) for the postsynaptic component (Tsetsenis et al., 2014). Another method for visualizing neuroligin-neurexin interactions is based on mutated bacterial lipoic acid ligase (LpIA) and lipoic acid acceptor peptide (LAP) tags (Uttamapinant et al., 2010, 2012), and is called Interaction-Dependent Probe Incorporation Mediated by Enzymes (ID-PRIME; Liu et al., 2013). Using the same principle of SynView, ID-PRIME was designed to detect trans-synaptic contacts of neurexin and neuroligin enzymatically using lipoic acid. These two methods successfully imaged the direct interactions of neurexin-neuroligin synaptic adhesive molecules. However, these methods seem restricted to particular investigations of the dynamics of neurexin-neuroligin, rather general synaptic mapping. In addition, it is known that overexpressing these synaptic adhesion molecules causes substantial structural and physiological perturbations of normal synaptic compartments and cleft structures.

## Bridging Tools Between Molecular Synapse Detectors and Brain-Wide Synapse Mapping

**Figure 3:**
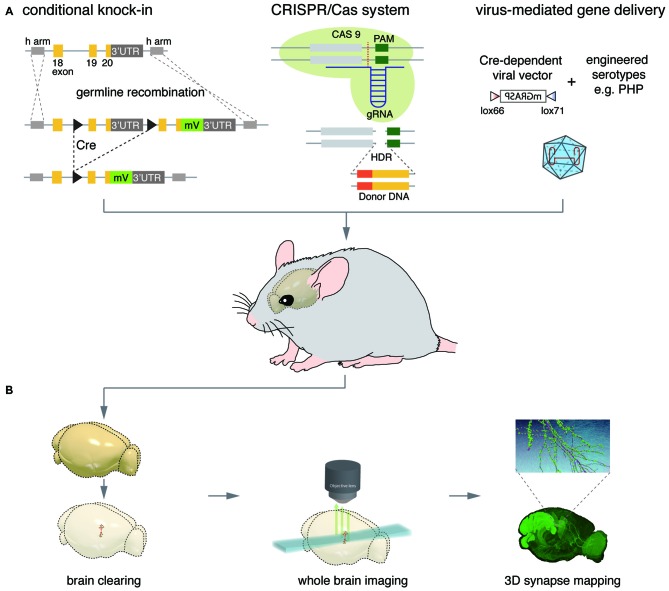
**Convergence of technologies for synaptic neuroscience at the systems level. (A)** Synapse-detector genes can be delivered to target neural components through germline manipulation-based and viral system-based methods. Exogenous genetic materials can be effectively introduced to the germline with high expression specificity via Cre-mediated conditional knock-in (KI) strategies and the homology directed repair (HDR) pathway of the clustered regularly-interspaced short palindromic repeats (CRISPR)/Cas system. In a Cre-mediated conditional KI strategy called endogenous labeling via exon duplication (ENABLED), a KI cassette including duplicates of exons 19 and 20 and the 3’UTR of PSD-95 is generated. The first duplicate is flanked by head-to-tail oriented loxP sites (black arrows), while the second duplicate has monovalent Venus (mV) inserted between exon 20 and the 3’UTR. The two duplicate sequences, along with exon 18, are flanked by a set of homology arms (h arm), which mediates KI cassette insertion through homologous recombination. Cre-lox recombination excises the first duplicate containing translation stop signals and polyadenylation sequences, and activates mV expression only in Cre-expressing neurons. Transient expression of sensor genes using viral vectors benefits from efforts to engineer expression through Cre-dependent expression cassettes e.g., Cre-dependent Mammalian GRASP (mGRASP) constructs and newly engineered serotypes that have more selective tropism and transduction efficiency (e.g., AAV-PHP.B). Local tissue or systemic injection of such viral systems can lead to flexible, versatile gene delivery in mature organisms. **(B)** Combination of light-favoring brain clearing, whole-brain imaging, and computational techniques for three-dimensional synapse mapping enables single-synapse level analysis of synaptic profiles across the whole brain. Further improvements in lipid extraction, refractive index matching, advanced light-sheet microscopy, and large-scale data processing and 3D reference space generation will accelerate systems neuroscience at the synaptic scale.

Thus far, we have described methods for detecting synapses focusing on molecular engineering. To exert these genetically encoded synapse detectors to brain-wide synaptic mapping at the system level, there need to be critically partnered technologies such as gene delivery, advanced imaging, and digital representation platform that are appropriate for neural system (Figure 3).

### Gene Delivery System for Synapse Detectors in the Mouse Brain

Improving the targeting specificity for types of cells and scaling up the scope of synaptic sensor delivery are among the crucial initial steps needed to expand single-synapse level analysis to systems level neuroscience. Gene delivery technologies, particularly for the nervous system, have developed at a breathtaking pace over the past decades, establishing methodologies that can be classified into two main categories: germ line manipulation and viral injection.

#### Genetic Manipulation-Based Gene Delivery

To avoid side effects that can sometimes be caused by the overexpression of FP-tagged synaptic proteins, and to mimic expression patterns of endogenous proteins, gene knock-in (KI) technology has been used to substitute wild-type genes with FP-tagged copies. For synaptic detectors based on universal synaptic proteins such as PSD-95, however, this standard KI method leads to expression of FP-tagged synapse detectors globally, throughout the brain, which makes it difficult to acquire high resolution images of a particular cell type. Therefore, a recent study introduced a conditional KI strategy called endogenous labeling via exon duplication (ENABLED; Fortin et al., 2014). In the ENABLED strategy, a knocked-in gene cassette is composed of the floxed last exon and 3’UTR of PSD-95 followed by its mVenus-tagged duplicated last exon and 3’UTR. The FP-tagged duplicated gene will be selectively expressed only in Cre-expressing neurons because its translation is designed to be blocked by translation stop signals in the endogenous copy in the absence of Cre recombinase. When this PSD-95-ENABLED mouse line is crossed with a dopaminergic cell-type specific DAT-Cre line, for instance, PSD-95^mVenus^ is expressed specifically in dopaminergic neurons. PSD-95-ENABLED was shown to functionally replace wild-type PSD-95 and to sparsely label a particular cell-type, allowing insights into the detailed distribution and dynamics of synapses. In applying this strategy to a wide variety of synaptic proteins, one concern with using the ENABLED strategy is that it is limited to synaptic proteins that are compatible with C-terminal FP-tagging.

Thanks to incredibly fast developments and improvements in genetic manipulation technologies such as clustered regularly-interspaced short palindromic repeats (CRISPR) and effector nucleases Cas system (Cong et al., 2013; Wang et al., 2013; Fujii et al., 2014; Aida et al., 2015), the generation of synapse detector KI mouse lines is becoming time- and cost-efficient, and is dramatically facilitating synapse mapping.

#### Viral System-Based Gene Delivery

Spatially targeted gene delivery into the mature brain can be achieved through stereotactic microinjection of viral vectors expressing FP-tagged proteins. Diverse virus families have been recruited into this effort: Retroviridae (e.g., lentivirus), Parvoviridae (e.g., rAAV), Adenoviridae (e.g., canine adenovirus), and Alphaviridae (e.g., sindbis virus), including some that cross the synapse, such as Rhabdoviridae (e.g., rabies virus) and Herpesviridae (e.g., HSV-1 and pseudorabies virus; Nassi et al., 2015). Because of their low cytotoxicity and stable expression, lentiviruses and rAAVs are the most widely used viral vectors in neuroanatomical tracing studies and human clinical trials testing gene therapy. In fact, spatially restricted injection of Cre-(in)dependent rAAV vectors have been used for mGRASP-assisted synaptic mapping.

In parallel, ongoing efforts include searching for and engineering new types of virus to allow transduction efficiency/specificity and retrograde infection. Canine adenovirus has drawn attention because of its strong retrograde transport capability and relatively large payload size (~30 kb); further, several successful applications of Canine adenovirus in the mouse brain suggest a powerful complement to the lentivirus and rAAV (Bru et al., 2010; Ekstrand et al., 2014; Junyent and Kremer, 2015; Schwarz et al., 2015). Additionally, systemic delivery of viral vectors in animal models has proved to be effective and safe for various serotypes of AAV (Foust et al., 2009; Bevan et al., 2011; Gray et al., 2011; Yang et al., 2014; Deverman et al., 2016).

### Advanced Brain-Wide Imaging and Digital Representation of Synaptic Detectors

Once FP-based synaptic detectors are introduced into the brain as described above, appropriate brain-wide imaging and digital representation technologies are necessary to map synaptic connectivity. Happily, there has been remarkable progress in tissue clearing methodology such as BABB (Dodt et al., 2007), CUBIC (Susaki et al., 2014) 3DISCO (Ertürk et al., 2012), immunolabeling-enabled three-dimensional imaging of solvent-cleared organs (iDISCO; Renier et al., 2014), SeeDB (Ke et al., 2013) and CLARITY (Chung et al., 2013; Tomer et al., 2014) that are needed to prepare the intact brain for imaging while avoiding distortions caused by physical sectioning. These new clearing techniques, together with advanced optical methods such as fluorescent selective plane illumination microscopy (fSPIM; Huisken et al., 2004), will allow high-throughput whole brain imaging. Also, once images are acquired, digital representation programs are necessary to reliably extract synaptic wiring information from the images, and to bring data from different sections and animals into register with one another (Johnson et al., 2010; Oh et al., 2014). Improvements in this software will be greatly beneficial.

In our view, new clearing methods, optics, and tailored computational analysis platforms together with advanced synaptic detectors such as mGRASP are very promising developments for the complete mapping of mammalian synaptic connectivity.

## Conclusion and Perspective

Here we reviewed new FP-based synapse detection techniques, which are useful for rapidly imaging individual synapses and synaptic connectivity in the whole brain with LM. Recent technical developments have allowed a focus of neuroscience to move from individual synapses to ensemble interactions among neurons of various cell types through multiple synaptic pathways. Comprehensively mapping individual synapses in the whole brain will provide firm grounds for further anatomical and functional studies and such mapping is essential for analyzing large-scale information processing phenomena. We believe that rapid, scalable synaptic cartography with the triad of single-synapse resolution, brain-wide scope, and cell-type-specific connectivity requires a synergistic combination of advanced technologies, and that FP-based neurosensors are the key to this grand integrative project.

## Author Contributions

HL, WCO, JS, and JK wrote this manuscript.

## Conflict of Interest Statement

The authors declare that the research was conducted in the absence of any commercial or financial relationships that could be construed as a potential conflict of interest.

## Abbreviations

3′UTR: 3′ untranslated region
AMPAR: α-amino-hydroxy-5-methyl-4-isoxazolepropionic acid receptor
CA3: Cornu Ammonis 3
CALI: chromophore-assisted light inactivation
CaMKII: calcium/calmodulin-dependent kinase II
CFP: Cyan fluorescent protein
CD4: cluster of differentiation 4
ChR: Channelrhodopsin
CRISPR: clustered regularly-interspaced short palindromic repeats
ENABLED: endogenous labeling via exon duplication
EM: electron microscopy
FMN: flavin mononucleotide
FP: fluorescent protein
fSPIM: fluorescent selective plane illumination microscopy
GFP: green fluorescent protein
HA: hemagglutinin
HSV-1: Herpes simplex virus-1
iDISCO: immunolabeling-enabled three-dimensional imaging of solvent-cleared organs
ID-PRIME: Interaction-Dependent Probe Incorporation Mediated by Enzymes
InSynC: Inhibition of Synaptic Release with CALI
KI: knock-in
LAP: lipoic acid acceptor peptide
LM: light microscopy
LOV2: light, oxygen, and voltage 2
LpIA: lipoic acid ligase
mGluR: metabotropic glutamate receptors
mGRASP: Mammalian GFP Reconstitution Across Synaptic Partners
miniSOG: mini small singlet oxygen generator
NCBI: National Center for Biotechnology Information
NS3: Nonstructural protein 3
OFP: Orange fluorescent protein
PDZ: PSD-95, Drosophila disc large tumor suppressor (Dlg1), and zonula occludens-1 protein (zo-1)
PSD-95: postsynaptic density protein-95
rAAV: recombinant adeno-associated virus
SM protein: Sec1/Munc18-like protein
SNARE: SNAP (Soluble N-ethylmaleimide-sensitive factor attachment protein) Receptor
syb: synaptobrevin
spGFP: split-GFP
sypHTomato: Synaptophysin-fused pHTomato
TimeSTAMP: Time-Specific Tagging for the Age Measurement of Proteins
VAMP2: vesicle-associated membrane protein 2
VenusNT: Venus N-terminal
VenusCT: Venus C-terminal
VGluT: vesicular glutamate transporter
YFP: Yellow fluorescent protein.
